# An epithelial cell culture model for sturgeon integument responds sensitively to 2,3,7,8-tetrachlorodibenzo-p-dioxin exposure

**DOI:** 10.1038/s41598-025-12299-7

**Published:** 2025-07-24

**Authors:** Sumi Nechat, Melina Shadi, Andrea D. Schreier, Nann A. Fangue, John P. Sundberg, Robert H. Rice

**Affiliations:** 1 Department of Environmental Toxicology, University of California, Davis, CA 95616 USA; 2 Department of Animal Science, University of California, Davis, CA 95616 USA; 3 Department of Wildlife, Fish and Conservation Biology, University of California, Davis, CA 95616 USA; 4 The Jackson Laboratory, Bar Harbor, ME 04679 USA; 5 Department of Dermatology, Vanderbilt University Medical Center, Nashville, TN 37232 USA

**Keywords:** AHR ligand binding model, Epithelial histology, EROD induction, Keratin immunostaining and content, PacBio RNA sequencing, Cell biology, Environmental sciences, Pathogenesis

## Abstract

Sturgeon species are threatened or endangered worldwide due to habitat loss and increasing pollution. An epithelial cell culture model promises to help investigate hazardous environmental exposures. Stratified squamous integument of green and white sturgeons (*Acipenser medirostris* and *transmontanus*, respectively) and cells cultured from their epithelia expressed substantial levels of TGM1-like transglutaminases, types I and II keratins and aryl hydrocarbon receptor (AHR) proteins analogous to those in mammalian integument. Epithelial cells cultured from the rim of the mouth, oral cavity and ampullae of Lorenzini exhibited dramatic growth suppression upon exposure to environmentally relevant levels of 2,3,7,8-tetrachlorodibenzo-p-dioxin (TCDD), a model compound for ubiquitous environmental combustion products that activate the AHR. The rho kinase inhibitor Y27632 markedly increased cell growth in culture and, surprisingly, prevented growth suppression by TCDD. Thus, these epithelial cell cultures provide an intriguing model for studying the mechanism by which activation of the AHR produces adverse effects. Evidently, contributions from genome duplication, gene duplication, gene loss and complications of polyploidization have endowed sturgeon with multiple forms of AHR, in this case 3 pairs of closely related genes. AHR1 and AHR2 are predicted to bind TCDD with high affinity, which could help rationalize the cellular sensitivity to AHR activation.

**Supplementary Information:**

The online version contains supplementary material available at 10.1038/s41598-025-12299-7.

## Introduction

Major commercial fisheries are in world-wide decline from pollution of the aquatic ecosystem^1^, intensive harvesting, loss and degradation of habitat^2^ and climate change^3^. Efforts to improve environmental protection overall, to restore degraded habitat and to maintain sustainable fish populations are dependent on understanding and ultimately regulating or mitigating many factors that have led to their dramatic declines. With respect to pollution, toxicology testing using multiple species with a diversity of sensitivities is anticipated to improve identification of specific pollutants of greatest importance^4^. This goal will be facilitated by fast and inexpensive mechanistic testing adaptable to high throughput formats using cell-based and in vitro systems rather than live animals for environmental as well as human hazard assessments^5^.

Among the many fish species currently threatened and endangered worldwide are sturgeon, which are threatened by historical overharvest for caviar and by impoundment and degradation of their feeding and spawning habitats. For example, the San Francisco Bay and Delta comprise an ecosystem where sturgeon and a variety of other species live or spend part of their life cycle. This estuary suffers from pollution at hazardous levels by metals, pesticides and persistent chlorinated aromatic and fluorinated hydrocarbons (including chlorinated dioxins and furans) that threaten the ability of aquatic fauna to thrive^6^. Atlantic (*Acipenser oxyrinchus*), shortnose (*A. brevirostrum*) and lake sturgeon (*A. fulvescens*) are quite sensitive in early life stages to developmental effects from the persistent chlorinated aromatics 2,3,7,8-tetrachlorodibenzo-p-dioxin (TCDD) and polychlorinated biphenyl 126, to which they are exposed at environmentally relevant levels in habitats outside California^7,8^. Based on transcriptional data from liver explants, white sturgeon (*A. transmontanus*) have been predicted to be more sensitive than lake sturgeon to TCDD and possibly as sensitive as rainbow trout (*Oncorhynchus mykiss*)^9^. Present work explores the sensitivities to TCDD of epithelial cells cultured from two species of special interest in California, white and green sturgeon (*A. transmontanus* and *A. medirostris*). Such cultures promise to facilitate toxicity testing while minimizing use of live animals.

Sturgeon are of intrinsic interest as “living fossils” that have changed little in morphology and physiology since they first appeared in the fossil record some 200 million years ago^10^. The very slow rate of germline mutation among sturgeon^11^ raises the question of how different are green and white sturgeon from each other, diverging about 100 million years ago, and from the vertebrate lineage from which they diverged nearly 350 million years ago. Our previous study of the properties of cells cultured from the integument of these two species revealed certain elements in common with, and others distinct from, human epidermal keratinocytes, providing insight into a possible evolutionary pathway permitting terrestrial adaptation^12^. Present work exploits transcriptional analysis with PacBio sequencing of mRNA expressed by cultured cells and the tissues from which cultures were derived. Initial results suggest these cultures adopt a similar keratinocyte-like phenotype, emphasizing the value of studying sturgeon for evolutionary insights.

## Materials and methods

### Sample collection

Green and white sturgeon tissue samples were collected from broodstock fish (no experimental treatments) housed at the Center for Aquatic Biology and Aquaculture, University of California, Davis. White sturgeon samples were also collected from Sterling Caviar (Elverta, CA) through the courtesy of Joshua Lang. Various epithelial tissues, such as oral mucosa (upper palate), esophagus, barbel, rim of the protrusible mouth and regions of epidermis were dissected from sturgeons and transported to the lab in culture medium at ambient temperature. The tissues were divided into two sets for each fish. One set was used for culturing primary cells and isolating RNA, while the other set was used for performing histology, immunohistochemistry (IHC) and laser-capture microdissection. Until sacrifice, sturgeon obtained at the University of California Davis were maintained in strict accordance with the recommendations in the Guide for the Care and Use of Laboratory Animals of the National Institutes of Health using IACUC protocols #19,778 (white) and #20,968 (green) approved by the campus Institutional Animal Care and Use Committee. Individual sturgeon were euthanized prior to sampling using buffered tricaine methane sulfonate. Some samples of white sturgeon were dissected from heads provided by Sterling Caviar immediately after sacrifice for production of caviar.

### Cell culture

Dissected tissues were rinsed well with 0.5 mM EDTA in phosphate buffered saline (PBS) to eliminate blood and other contaminants. The primary sturgeon cells were cultured as explants before (white) or after trypsinization (green) in 6 cm dishes to optimize tissue attachment and epithelial outgrowths as previously described for tilapia (*Oreochromis niloticus*) and sturgeon^12,13^. Cultures were maintained at 22–25 °C as previously described in a 2:1 mixture of high glucose Dulbecco-Vogt modified Eagle’s and F12 media supplemented with fetal bovine serum (5%), epidermal growth factor (10 ng/ml), hydrocortisone (0.4 µg/ml), Y27632 rho kinase inhibitor (10 µM), transferrin (5 µg/ml), insulin (5 µg/ml), penicillin (100 units/ml) and streptomycin (100 µg/ml). Ciprofloxacin (10 µM) and amphotericin B (1–2 µg/ml) were initially used for several medium changes to prevent microbial growth. The media were changed every 4 days until the cultures became confluent when the cells were passaged (typically 1:2) or harvested for rhodanile blue staining and immunocytochemistry (ICC). From various explants, some lost to contamination, epithelial cultures were established from green sturgeon upper palate (GSI) and ampullae of Lorenzini (GSA) and from white sturgeon mouth rim (WSM). Fibroblasts (WSF) were also cultured from mouth rim tissue. After several passages as above, subculturing was performed at high density (typically 1:2) in Leibowitz L-15 medium containing 5% fetal bovine serum and either 10 µM Y27632 for epithelial cells or 5 µg/ml insulin for fibroblasts.

To assess the effect of various additives on their responses, cells were seeded in 12 well plates in L-15 medium (1 ml per well) supplemented with 5% fetal bovine serum. At each medium change (3 day intervals), 1 µl of each factor of interest was added per well from a sterile stock solution in aqueous solution (EGF, insulin) or in dimethylsulfoxide (Y23762, TCDD) or a solvent control. For measurement of growth, the wells were rinsed twice in phosphate buffered saline and harvested in 0.5–0.8 ml of 1% SDS. The protein content was measured using bicinchoninic acid^14^ with bovine serum albumin as standard. Confluent wells contained ≈ 300 µg of protein. For measurement of ethoxyresorufin-O-deethylase (EROD) activity^12^, a measure of induced cytochrome P450 1A activity, the medium was removed and wells were incubated for 2 h in 1 ml of L-15 medium containing 7-ethoxyresorufin (4 µM). Fluorescence was then measured (excitation at 560 nm, emission at 600 nm) using a Molecular Devices iD3 fluorometer, where 1 µM resorufin product gave 9.2 × 10^5^ fluorescence units.

### Histology, immunohisto-and immunocytochemistry (IHC and ICC)

Dissected white sturgeon tissues were fixed by immersion in Fekete’s acid-alcohol-formalin for not more than 24 h^15^washed twice with cold PBS and transferred to 70% ethanol. Tissues were infiltrated with paraffin and processed (Tissue-Tek VIP Vacuum Infiltration Processor, Sakura). Sections of 4 μm were stained with hematoxylin and eosin (H&E) for routine evaluation or alcian blue/periodic acid Schiff (PAS) reagent at pH 2.5 to visualize mucus production^16^. For immunohistochemistry, unstained serial sections were treated with a monoclonal mouse antibody pan-cytokeratin AE1/AE3 cocktail (Sigma-Aldrich) that detects numerous acidic type I and basic type II mammalian keratins^17^ and processed as described^18,19^. Briefly, sections were blocked with bovine serum albumin (BSA), incubated overnight at 4 °C with the primary antibody (1:100 dilution), washed and incubated with anti-mouse IgG HRP-linked antibody (Cell Signaling Technology) for 1 h, followed by diaminobenzidine staining for 5 min or until brown color development, with PBS rinsing to stop the reaction. Slides were also labeled with anti-pan-cytokeratin PCK-26 antibody (Abcam 6401) diluted 1:800 with no antigen retrieval using a Bond RX Leica automatic stainer. Immunocytochemistry (ICC) was similarly performed using the AE1/AE3 cocktail on cultured epithelial cells on gelatin-coated slides with minor adjustments. Cells were permeabilized with 0.5% Tween 20 in PBS, blocked with 1% BSA in Tween-PBS, and incubated overnight at 4 °C with the primary antibody (1:100). After washing, they were incubated with HRP-conjugated secondary antibody (1:500), followed by DAB staining. Sections of sturgeon tissues all were immunopositive for keratin in this way, and control sections lacking primary antibody were immunonegative (example of oral tissue shown in Supplementary Fig S1). Cells cultured from green sturgeon were stained with rhodanile blue^20,21^ and, essentially as described for white sturgeon, with anti-pan-cytokertain AE1/AE3. Microscopic images were captured using an Olympus BX50 microscope with a DP27 digital camera for the IHC and a BH2-RFCA microscope for the ICC.

### Laser-capture microdissection

Epithelial tissues from green sturgeon were diced into small pieces with single edged razor blades and placed in cryomolds with Optimal Cutting Temperature (OCT) compound. Tissues were frozen in 2-methylbutane over liquid nitrogen, and 10 μm sections were cut using a Leica CM1850 cryostat at -20 °C. The sections were mounted on PEN-Membrane slides (Leica) for laser-capture microdissection. Selected tissue areas (1 to 10 million µm²) from each sample were pooled and stored at -80 °C until RNA extraction, as previously described^22^.

### RNA extraction, purification and sequencing

Primary cells from white and green sturgeon cultures and epithelial tissues were collected in 700 µL of RLT Plus lysis buffer (Qiagen) and stored at -80 °C prior to RNA isolation. Dissected white sturgeon tissues were disrupted in RLT Plus buffer using an Omni Bead Ruptor 24. RNA was extracted from cultured epithelial cells, tissue lysates, and laser-microdissected samples using the RNeasy Plus Mini Kit (Qiagen) with gDNA eliminator columns. Genomic DNA was removed, and RNA was further purified with the RNA Clean & Concentrator-5 DNase I Kit (Zymo Research).

Total RNA from cultured cells and tissue samples was used for PacBio sequencing by the UC Davis DNA Technologies Core. cDNA and SMRTbell libraries were constructed using 300 ng of total RNA with the NEBNext^®^ Single Cell/Low Input cDNA Synthesis & Amplification Module (New England Biolabs). After 15 cycles of PCR amplification, cDNA was purified using 0.86X SMRTbell cleanup beads. cDNA libraries were pooled for SMRTbell library construction using the SMRTbell prep kit 2.0 or 3.0 (Pacific Biosciences, Menlo Park, CA) with repair and A-tailing, overhang adapter ligation, and nuclease treatment. The Iso-Seq SMRTbell library was sequenced at UC Davis DNA Technologies and Expression Analysis Core (Davis, CA) using one 8 M SMRT cell (Pacific Biosciences, Menlo Park, CA; Cat #101-389-001), Sequel II sequencing chemistry 2.0, and 24-hour movies on a PacBio Sequel II sequencer. cDNA preparation, amplification, and library prep were performed with the Iso-Seq Express Oligo Kit (PacBio) and SMRTbell Prep Kit 3.0 (PacBio).

Iso-Seq data analysis and gene annotation were completed by the UC Davis Bioinformatics Core. Raw sequencing data were analyzed according to the IsoSeq3 pipeline (v3.4.0, https://isoseq.how/*)* and aligned to the sterlet genome (*Acipenser ruthenus*, ASM1064508v1)^23^. Resulting transcripts were classified and filtered using the default criteria within Sqanti3 (v4.1)^24^ with the exception of the splice junction coverage filter, which was replaced by a minimum supporting reads filter ( > = 3). The command line implementation of blastp (v2.14.1+)^25^ was used to align the translated transcripts to the protein sequences of the sterlet genome for functional annotation. Alignments with an e-value less than 0.01 and at least 80% sequence identity were considered significant.

### Quantitative real time PCR and gene expression

Quantitative real time PCR (qPCR) was employed to find relative expression levels of genes anticipated to be relevant for keratinocyte character or toxic responses (transglutaminase 1, keratin, cytochrome P450, aryl hydrocarbon receptor, sulfhydryl oxidase) or for normalization (desmoplakin, filamin A). PacBio-derived RNA sequences were used to derive alignments from which specific primer sets could be deduced. (The primer set for transglutaminase 1 was designed in this way and used previously^12^.) For each transcript, regions were selected with identical sequences for both species for primer design. Keratin sequences were divided into groups I and II most closely related to acidic and basic types, respectively. Similarly, single primer sets were used for mRNAs from *Ahr1* (a and b, PacBio transcripts observed in similar numbers), for *Ahr2* (a and b, PacBio transcripts observed similar numbers) and *Ahr4*. Based on such alignments, custom Taqman assays for 12 sturgeon mRNAs were purchased from Applied Biosystems Life Technologies. Primer and reporter nucleotide sequences are given in Supplementary Table S1. Total RNA (2 µg) from cultured epithelial cells, tissue samples, and laser-microdissected tissues was reverse transcribed into cDNA using a reverse transcription kit (Applied Biosystems) and treated with recombinant DNase (Ambion Turbo DNAse-free kit) to remove contaminating DNA. qPCR was performed with Taqman assays using an Applied Biosystems QuantStudio3 instrument, and relative gene expression levels were calculated by the comparative CT (2^−ΔΔCT^) method^26^where the root mean square of transcripts from the reference sturgeon genes filamin A (*Flna*) and desmoplakin (*Dsp*) was used for normalization.

### Protein measurement, SDS-PAGE and Immunoblotting

Each confluent culture was rinsed twice in PBS, harvested in 1 ml of 0.6 M KCl − 1% triton X-100–0.05 M Tris buffer (pH 7.5), centrifuged 3 min at 10,000 x g and extracted a second time. The pellet was then sonicated in 1 ml of the Tris - Triton buffer without KCl and extracted a second time. The pellet, consisting of keratin that resists solubilization in Triton X-100^27^, was dissolved overnight in 0.5–0.8 ml of 1% SDS with warming. The protein content of each fraction was measured. Samples of equal protein content were compared in 10% gels by SDS-PAGE with Coomassie G-250 staining.

For western blot analysis, separated proteins in gels were blotted onto Immobilon-P membranes (Millipore), blocked with nonfat dry milk (5%) in TBST (25 mM Tris (pH 7.5), 150 mM NaCl, 0.05% Tween 20) for 1 h, washed several times with TBST and incubated with monoclonal mouse pan-cytokeratin cocktail AE1 and AE3 antibody (1:10,000) diluted in sodium azide (5% BSA in TBST and 0.02% sodium azide) overnight at 4 °C. The membranes were washed 4 times with TBST, incubated with anti-mouse IgG HRP-linked (1:5,000) diluted in the blocking buffer for 1 h, followed by 4 washes in TBST. Protein bands in the membrane were visualized with ECL2 reagents (ThermoFisher Scientific) using a Thermo Pierce MyECL imager. Molecular weights were estimated using the PageRuler Plus prestained protein ladder (ThermoFisher). Original uncropped images from PAGE and immunoblotting are shown in Supplementary Figure S6.

### Statistical analysis

Results from protein analyses were analyzed by ANOVA using Statistics Kingdom online calculator https://www.statskingdom.com/180Anova1way.html, which tests for data normality and provides Tukey HSD pairwise comparisons. Except as noted, each experiment was performed independently in triplicate at least twice, of which a representative result is illustrated.

## Results

**Fig. 1:**
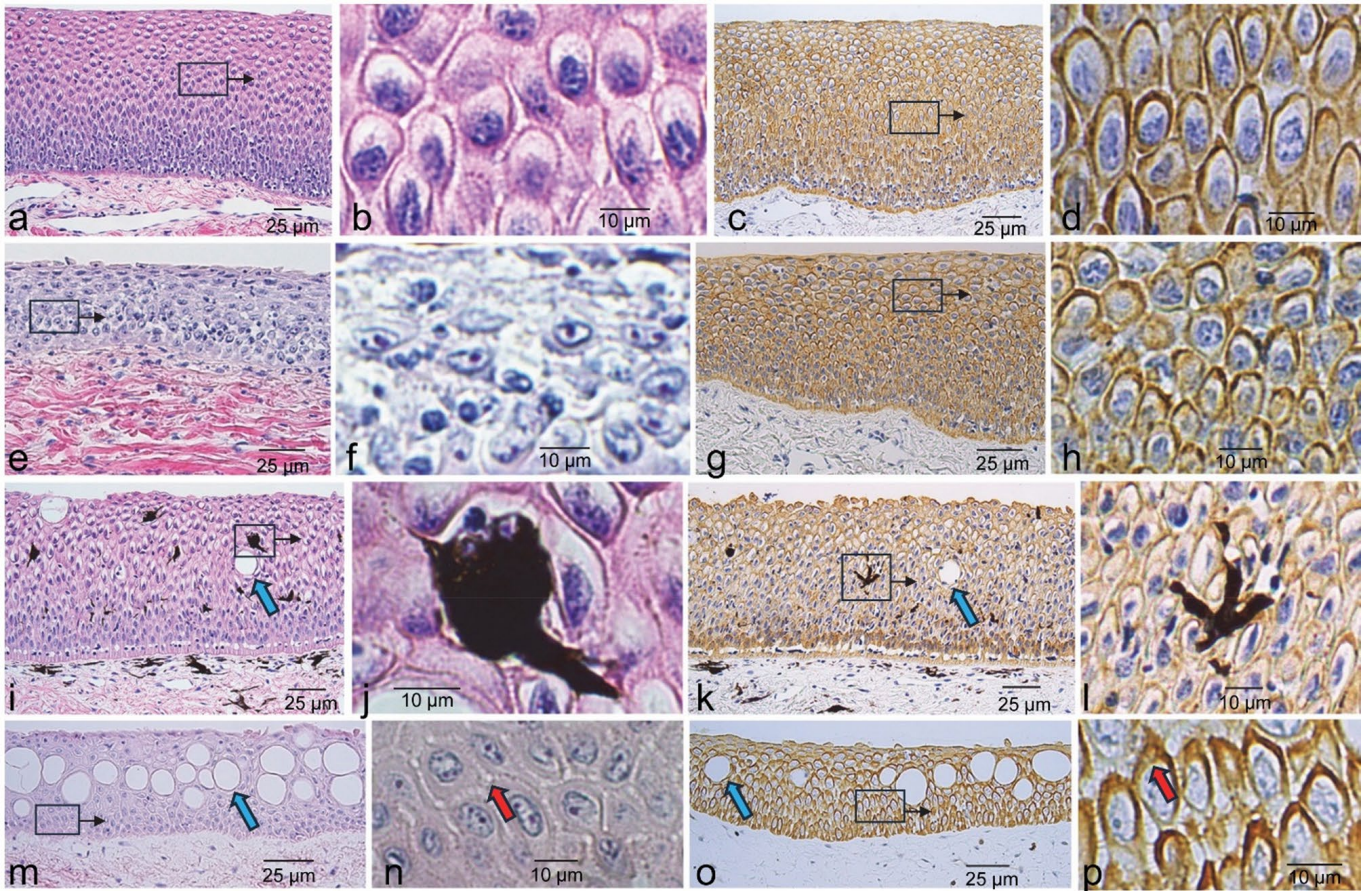
Sections of skin and epithelial tissues from white sturgeon. Samples are from the rim of the mouth (**a**–**d**), barbel (**e**–**h**), dorsal skin (**i**–**l**) and ampullae of Lorenzini (**m**–**p**). The left two columns show H&E staining, and the right two columns show keratin immunostaining. The second and fourth columns show enlargements of representative areas in panels to their immediate left at lower magnifications. Panels **j** and **l** show higher magnification of irregular, heavily pigmented dendrite-shaped cells characteristic of dorsal epidermis in (**i**, **k**), respectively. Blue arrows (**i**, **k**, **m**, **o**) point to round to oval spaces that lacked H&E and keratin staining. Red arrows point to apical regions of cells with “spines” indicative of desmosomal connections (**n**) and keratin accumulation (**p**).

Sturgeon skin does not have scales (although it covers bony plates called scutes in regions not sampled). It consists of the epidermis, dermis and hypodermis, while the mucus membranes have an epithelial layer, lamina propria, and underlying adventitia. The basic structures are similar with anatomic site-specific differences. The dorsal and ventral skin of white sturgeon, including rim of the mouth, epithelia of the barbels and ampullae of Lorenzini, were covered by non-cornifying stratified squamous epithelium seen at low (Fig. 1a, e, i, m) and higher magnification (Fig. 1b, f, j, n). Cuboidal to columnar cells formed the stratum basale on the basement membrane, a PAS-positive line at the junction of the epidermis and dermis. Above these, the cells became polyhedral with numerous “spines” between the cells, an artifact of shrinkage revealing desmosomal attachments as in the stratum spinosum of mammalian epidermis, evident at high magnification in both H&E and keratin IHC (Fig. 1d, h, l, p). The cells flattened parallel to the length of the skin forming a layer similar to the stratum granulosum in mammalian skin but without the keratohyalin granules. No stratum corneum was evident. The underlying dermis consisted of loose irregular collagenous connective tissue, blood vessels, and nerves. Scattered throughout the epidermis of the dorsal skin were irregular, heavily pigmented dendritic cells (Fig. 1i, j, k, l). Each epithelium displayed clearly positive cytoplasmic immunostaining with both pan-cytokeratin antibodies (Fig. 1c, g, k, o). In sections of the mouth (Fig. 1c), barbel (Fig. 1g) and dorsal skin (Fig. 1k), the keratin IHC revealed a clearly delineated basal layer.

**Fig. 2:**
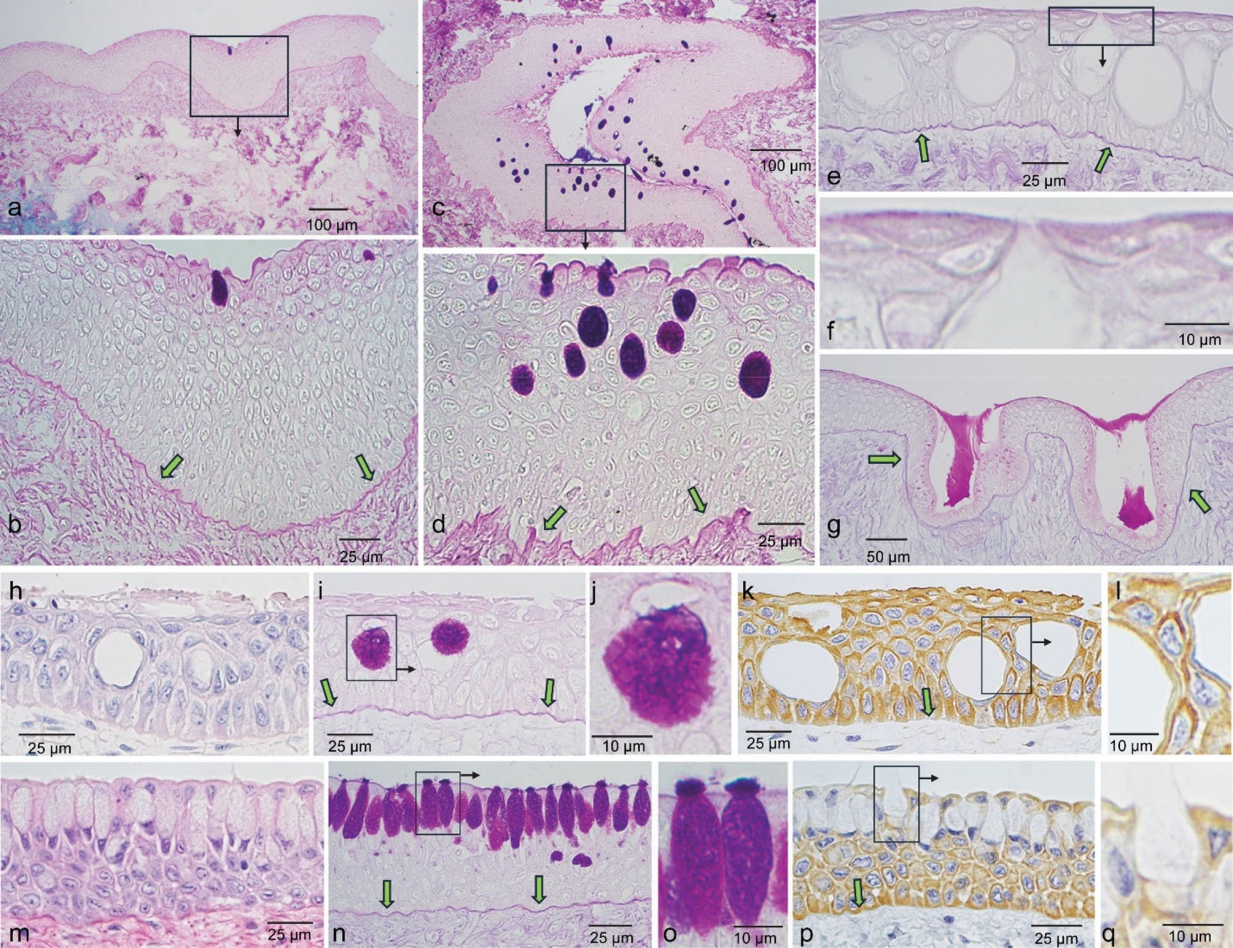
Sections labeled for mucin using PAS or for keratin with pan-cytokeratin antibodies. Shown are low and high magnification images of the rim of the mouth (**a**, **b**), oral tissue (**c**, **d**) and ampullae of Lorenzini (**e**–**g**). Panel (**g**) shows mucin-positive reaction of pit organs. Panels (**i**, **j**) (the latter at higher magnification) show mucin-positivity of the round or oval circles that appear empty in H&E staining (**h**) of ventral epidermis, while (**k**, **l**) show keratin immunoreactivity. Panels **n** and **o** (the latter at higher magnification) show mucin-positive reaction of apical cells in esophageal epithelium that appear empty by H&E (**m**) and keratin labeling (**p**, **q**). Green arrows point to a positive PAS reaction of the basement membrane in sections of mouth (**b**), oral epithelium (**d**), ampullae of Lorenzini (**e**, **g**), ventral skin (**i**), esophageal epithelium (**n**) and to the distinct boundary between the epithelium and the underlying supportive tissue in ventral skin (**k**) and esophagus (**p**).

Each epithelium had near round to oval spaces that lacked H&E and immunostaining, clearly visible in the dorsal skin and ampullae of Lorenzini in Fig. 1 (panels 1**i**, **k**, **m**, **o**). These features were observed at various densities, often appearing in clusters separating regions of low density. In sections of the rim of the mouth and oral cavity (upper palate), they reacted positively with PAS for mucin. A low density of mucus secreting cells is shown near the surface of the mouth epithelium (Fig. 2a, b), while clusters are shown in the mid to upper layers of the oral cavity (Fig. 2c, d). By contrast, these features, most prominent in the ampullae of Lorenzini, did not exhibit positive PAS reaction (Fig. 2e) even though some exhibited fissures leading to the epithelial surface (Fig. 2f). However, closely associated pit organs did exhibit positive PAS reaction (Fig. 2g).

The density of mucus secreting cells overall was considerably higher in the ventral than in the dorsal epidermis. PAS reactivity of two such secretory cells in sections of ventral skin (oval spaces not stained by H&E in 2**h**) are illustrated in Fig. 2i and one at higher magnification in Fig. 2j. The esophagus exhibited a stratified squamous epithelium with several layers of cuboidal epithelial cells on top of the basement membrane to form the stratum basale that differentiated into a stratum spinosum. The most superficial layer differentiated into tall columnar cells producing mucus (oval spaces unstained by H&E in 2**m**) that was secreted to the surface (Fig. 2n, o, p, q). Both epithelia were immunopositive for keratin (2**k**, **l**, **p**, **q**). In both, a well-defined basement membrane was revealed with the PAS reaction (Fig. 2i, n), observed also in sections of the mouth rim and ampullae of Lorenzini (Fig. 2b, d, e, g).

In parallel to tissue sections of white sturgeon above, epithelial cells cultured from green sturgeon were examined for evidence of keratin expression (Supplementary Figure S2). From the 7 anatomic sites examined (same as in white sturgeon), all stained red with rhodanile blue, presumptively staining for keratin-containing cells. Since this stain is not completely specific, the cells were also immunostained with pan-cytokeratin AE1/AE3 monoclonal antibodies. All the samples exhibited strongly positive cytoplasmic labeling.

To permit characterization of gene expression in the cell cultures, RNA preparations from the primary cultures and tissues of origin were subjected to PacBio Iso-Seq. The transcription products were then identified using as reference the sequences of sterlet sturgeon (*Acipenser ruthenus*)^23^. Since the sequences of green and white sturgeon transcripts exhibited high degrees of identity, regions of identical sequence were used to design each Taqman gene expression assay for a given gene (Supplementary Table S1). This design permitted a single assay to detect homologous transcripts from both species.

As previously described^12^epithelial cells were initially propagated with 3T3 feeder layer support under conditions optimized for growth of human epidermal cells^28^. These conditions are used rarely for fish cell culture, and the roles of added growth factors, chiefly epidermal growth factor (EGF), insulin and the rho kinase inhibitor Y27632, have not been characterized. For these reasons, cell culture conditions were explored before proceeding with characterization of cell strains cultured from the region of ampullae of Lorenzini (GSA) and upper palate of green sturgeon (GSI) and from the rim of the protrusible mouth of white sturgeon (WSM).

**Fig. 3:**
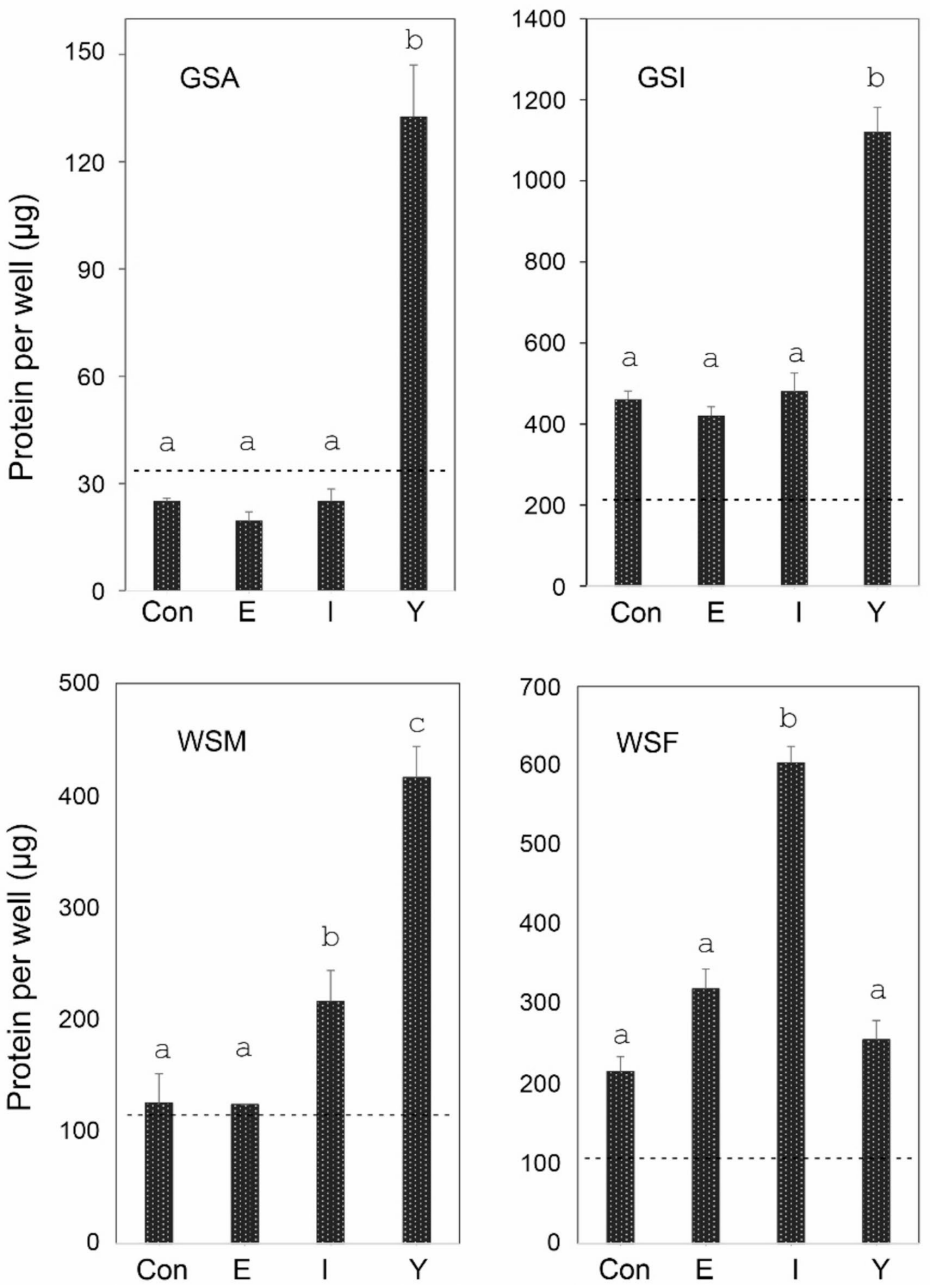
Cultures of the indicated cell strains (GSA, GSI, WSM, WSF) were maintained in medium containing 5% fetal bovine serum (Con) plus the indicated additives epidermal growth factor (E), insulin (I) or Y27632 (Y) for 15 days. The dashed lines indicate the protein content after one day when treatment began. Bars with the different letters above them were significantly different in value. GSA, *p* < 10^−7^; GSI, *p* < 10^−6^; WSM, a:b *p* = 0.03, a: c *p* = 10^−5^, b: c *p* = 10^−4^ ; WSF, *p* < 10^−3^.

After 5–7 initial passages, the cells were transitioned to L-15 medium containing 5% serum supplementation, and the influence of additives at concentrations optimal for stimulating human keratinocyte growth was measured. When the sturgeon epithelial cells (GSA, GSI, WSM) were subcultured in L-15 medium, their growth was stimulated markedly by Y27632 (Fig. 3). In such experiments, EGF and insulin exhibited small effects occasionally as shown but not reproducibly. By contrast, fibroblasts derived from the white sturgeon mouth tissue (WSF) were stimulated markedly by insulin, while Y27632 and EGF had little effect. These observations led to routine cultivation in L15 medium supplemented with 5% serum and either Y27632 (epithelial cells) or insulin (fibroblasts) except as noted.

**Fig. 4:**
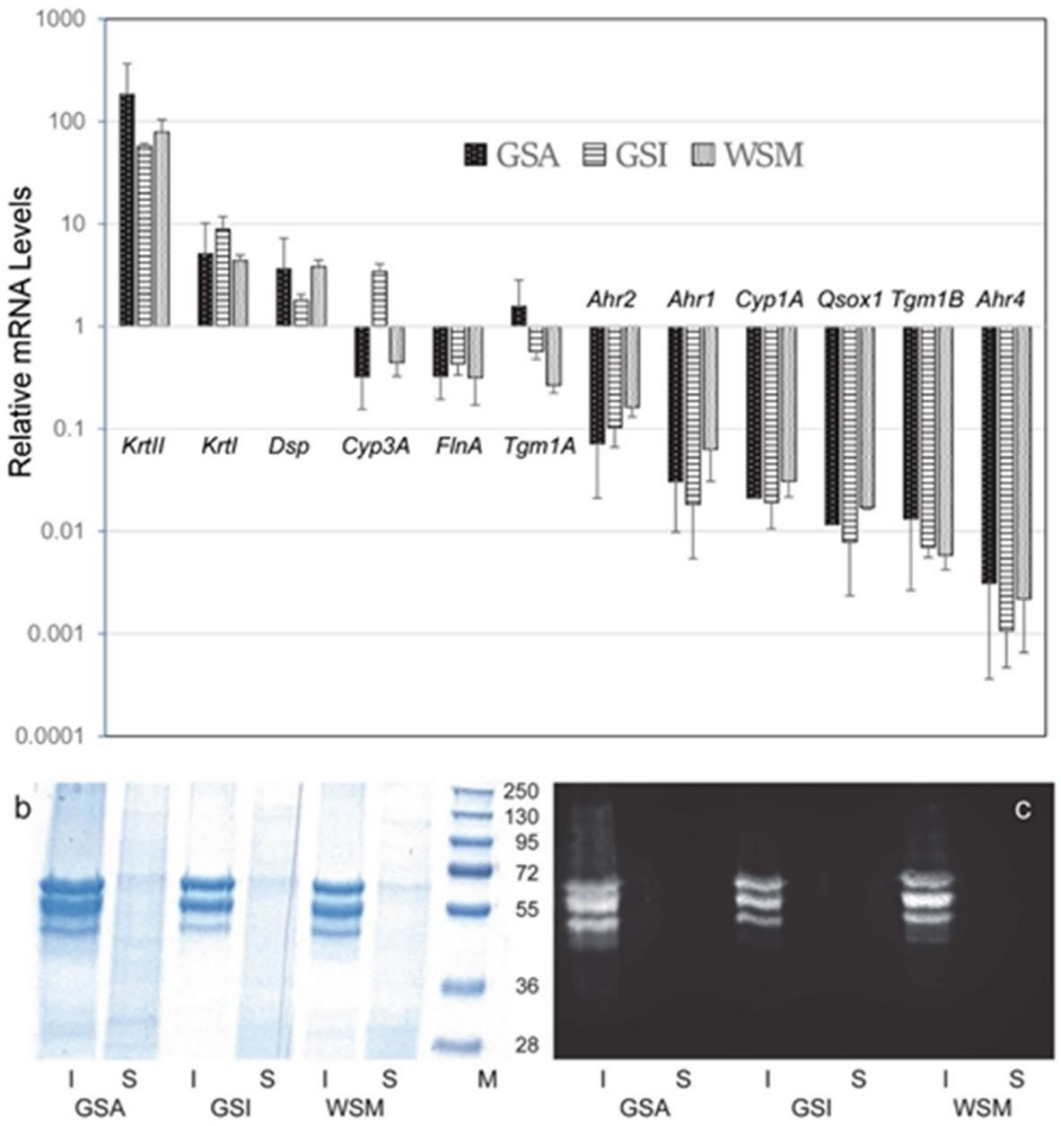
Evidence for relatively high levels of keratin expression in GSA, GSI and WSM cultures. (**a**) Relative amounts of mRNA transcribed from 12 genes are shown as determined by qPCR. The values from individual samples were normalized to the root mean square values for *Dsp* and *FlnA*. (**b**) Equal amounts of cell protein insoluble in (I) or solubilized by (S) Triton X-100 were compared in polyacrylamide gels stained with Coomassie G-250 in parallel to molecular weight marker proteins (M). (**c**) In parallel gels, equal amounts of the proteins were immunostained with pan-cytokeratin antibodies and visualized by chemiluminescence.

Previous work indicated that cultured sturgeon epithelial cells resembled mammalian keratinocytes in expression of two closely related transglutaminases, TGM1A and TGM1B, capable of forming cross-linked envelopes^12^. Strengthening the comparison to keratinocytes in present work, a relatively high level of keratin expression was also seen by analysis of mRNA levels using qPCR. For this measurement, keratin mRNAs were detectable using primers recognizing the acidic (I) and basic (II) keratin groups. As shown in Fig. 4a, the combined levels of keratin mRNAs were an order of magnitude higher in amount than those encoding other common proteins (DSP, FLNA). *Tgm1A* was expressed at levels comparable to *Dsp* and *FlnA*, while *Tgm1B* mRNA levels were nearly 100 fold lower. The semi-quantitative comparison of transcript numbers in the primary cultures versus tissues from which they were derived (ampullae of Lorenzini, esophagus, upper palate, rim of protrusible mouth, skin) revealed relatively high keratin levels in these cells (Supplementary Figure S3). The relative levels of keratins, *Tgm1A* and sulfhydryl oxidase (*Qsox1*) observed in transcript numbers were consistent with those seen by qPCR using mRNA from laser capture dissection-derived tissue and culture of ventral skin (Supplementary Figure S3).

**Fig. 5:**
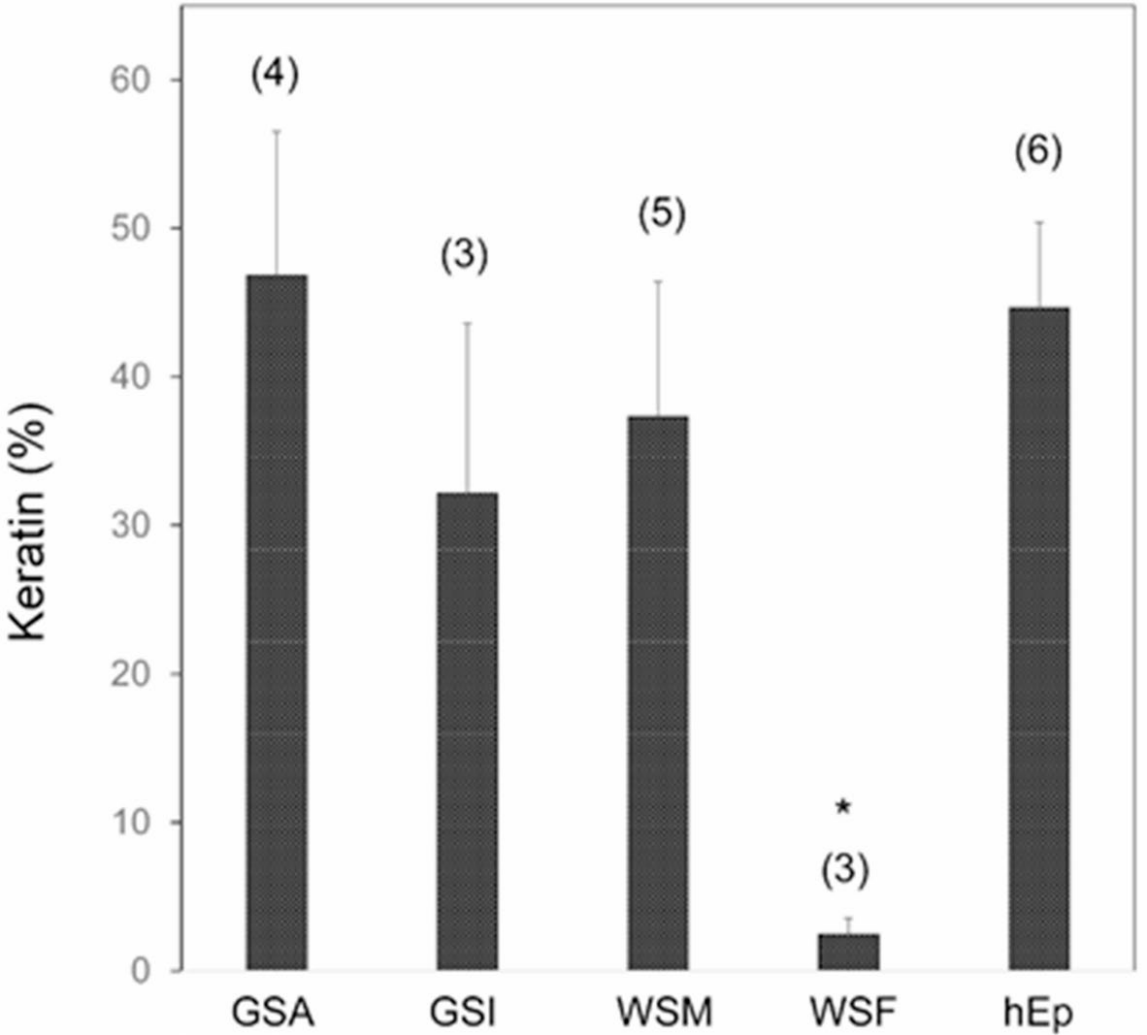
Keratin content estimated as Triton-insoluble protein. Samples were extracted from GSA, GSI and WSM epithelial cultures in parallel with WSF fibroblasts and human epidermal cultures (hEp). Above the bars are indicated the numbers of independent cultures analyzed. The value for WSF (*) was significantly lower than the others (*p* < 0.001), which did not differ significantly from each other.

Supporting the observation of abundant keratin mRNA in the epithelial cells, the insoluble protein from extracts displayed electrophoretic mobility in the molecular weight range of keratins and was immunoreactive with the pan-cytokeratin monoclonal antibodies. By contrast, the soluble protein appeared distributed over a wide range of mobilities and was not immunoreactive in this way (Fig. 4b, c). Measurement of the amount of keratin in the cells took advantage of their insolubility in Triton-X100. Extraction of the cultures in the three established strains (GSA, GSI, WSM) gave large fractions of insoluble protein. As shown in Fig. 5, a third or more of the cell protein from the epithelial cultures was in the insoluble fraction, whereas very little was insoluble from fibroblast cultures derived from white sturgeon mouth rim (WSF). In parallel cultures, human epidermal cells (grown using the 3T3 feeder layer system) gave essentially the same fraction of insoluble protein.

**Fig. 6:**
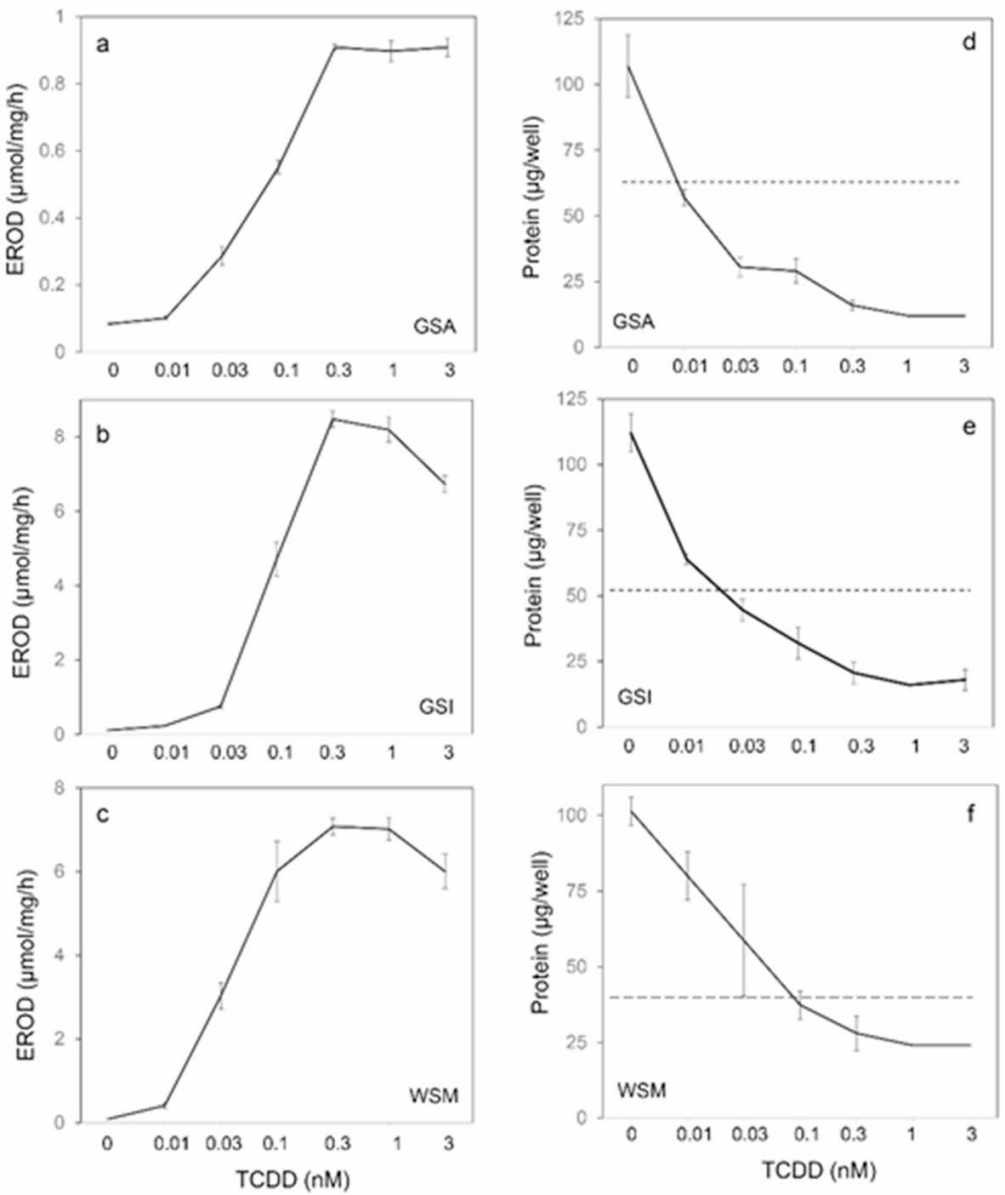
Dependence of CYP1A induction and growth suppression on indicated TCDD concentrations. (**a**–**c**) Induction of ethoxyresorufin-O-deethylase (EROD) in newly confluent cultures after overnight treatment (18 h) with the indicated concentrations of TCDD. (**d**–**f**) Suppression of growth after 12 (GSA), 10 (GSI) or 9 (WSM) days before harvesting. The dashed line shows the protein content of the wells at the start of treatment.

A second goal of this work was to find whether the cultured epithelial cells would be useful in understanding mechanisms of action of toxic chemicals in the aquatic environment. Since previous work showed that ethoxyresorufin-O-deethylase activity is inducible by TCDD in epithelial cells cultured from the rim of white sturgeon mouth^12^how well TCDD toxicity paralleled EROD induction was explored. First, the sensitivity of induction to TCDD concentration was measured in cell strains (GSA, GSI, WSM) of the three tissue origins. As shown in Fig. 6a, b, c, the EC50 for induction was between 0.03 and 0.1 nM in each case. Thus, TCDD was an order of magnitude more potent in the sturgeon epithelial cells than in cultured human epidermal cells assayed in this way (Fig S4). The extent of induction was quite similar in GSI and WSM cultures but substantially lower in GSA cells.

**Fig. 7:**
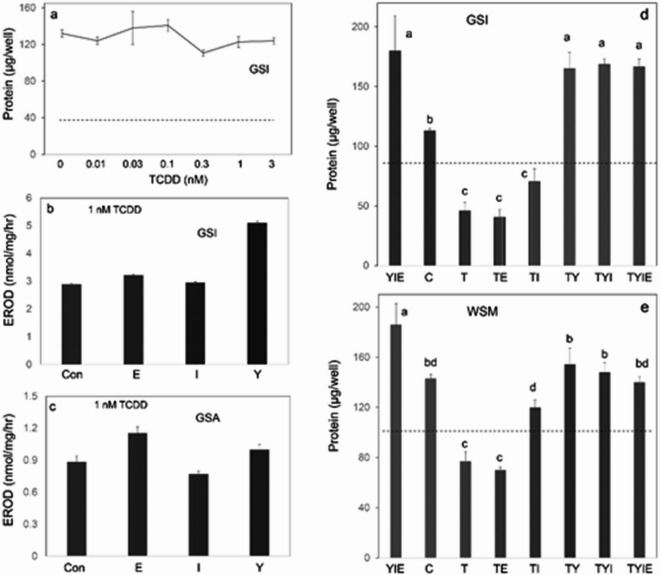
Effects of growth factors on cell responses to TCDD. (**a**) GSI cells displayed no suppression of growth in the presence of the medium additives EGF, insulin and Y27632 upon treatment with TCDD from 0.01-3 nM. At 1 nM, TCDD was similarly effective in inducing ethoxyresorufin-O-deethylase (EROD) activity in GSI (**b**) and GSA (**c**) cultures regardless whether they were grown in the absence (Con) or presence of EGF (E), insulin (I) or Y27632 (Y). Y27632 suppression of TCDD toxicity in GSI (**d**) and WSM (**e**) cells. One day after inoculation, cultures in 12 well plates were treated with 1 nM TCDD (T) either alone (Con) or singly with the growth factors Y27632 (Y), epidermal growth factor (E) or insulin (I) as indicated. The dashed lines show the protein content per well at the time treatment started. (**d**) GSI cells treated for 8 days; (**e**) WSM cells treated for 7 days. Values not significantly different from each other share letters above the bars; those with different letters were significantly different. **d**, a: b *p* < 2 × 10^− 4^, a: c *p* < 6 × 10^−9^, b: c *p* < 4 × 10^−3^. **e**, a: b *p* = 0.01, a: c *p* = 2 × 10^−9^, a: d *p* = 2 × 10^−6^, a: bd *p* = 3 × 10^−4^, c: bd *p* < 4 × 10^−6^.

The sensitivity of the epithelial cells to growth suppression by TCDD was measured. Cytopathic effects (cell rounding and release into the medium) were seen by day 4. As shown in Fig. 6d, e, f, addition of TCDD to the medium in the absence of Y27632 prevented cell growth or reduced the cell number in culture after a week of exposure even at the lowest concentration tested (0.01 nM). However, inclusion of growth factors EGF, insulin and Y37632 together permitted GSI cells to grow nearly as well as in the absence of TCDD (Fig. 7a). EROD activity was not obviously affected by these growth factors, since its induction was not impeded by them (Fig. 7b, c).

The toxicity of TCDD (cytopathic effects and suppression of growth) was examined when the medium was supplemented singly with EGF, insulin or Y27632 or in combinations. TCDD exhibited little if any toxicity when the medium was supplemented with Y27632 (Fig. 7d, e and Supplementary Fig. 5). In some experiments, insulin alone had a small positive effect on growth, but this response was not always seen, and it did not increase the effectiveness of Y27632.

The observed inducibility of *Cyp1A* and toxicity under certain conditions indicates the presence of high affinity aryl hydrocarbon receptor (AHR) in the cells. Inspection of AHR sequences obtained by PacBio Iso-Seq of white sturgeon transcripts revealed 5 distinct forms. Comparisons of translated sequences using NCBI BLAST showed the samples expressed two pairs of closely related sequences exhibiting 82–90% identity to each other but only 54–56% identity to the other pair (Supplementary Table S2). A fifth form (designated AHR4), expressed at very low levels, showed 46–49% sequence identity to the others. (As illustrated, green sturgeon AHR4a and AHR4b showed similar relationships to AHR1 and AHR2.) As shown in Supplementary Table S3, one pair corresponded to AHR1 and the other pair to AHR2 according to amino acid sequence comparisons with those reported previously for lake (*Acipenser fulvescens*) and white sturgeons^29^. Not clearly corresponding to either form by this criterion, AHR4 exhibited 46–49% identity to AHR1and AHR2.

Results from qPCR also revealed that *Ahr2* was expressed at several fold higher levels than *Ahr1* by all three cell lines, while *Ahr4* was detected at much lower levels. The possibility that *Ahr4* transcripts were not detected by PacBio Iso-Seq of green sturgeon RNA samples could be attributed to a much lower level of expression. Detection of *Ahr4* transcripts by qPCR in green sturgeon culture samples suggested that green sturgeon has the corresponding gene and the cells did express it. Using the white sturgeon *Ahr4* sequence to interrogate (BLASTn and tBLASTn) an in-house green sturgeon genomic scaffold revealed two closely related nearly full-length sequences corresponding to this protein. Using the white sturgeon AHR4 amino acid sequence to search (BLASTp) for matches in GenBank revealed two closely related sequences in sterlet (*A. ruthenus*), one in Atlantic sturgeon (*A. oxyrinchus oxyrinchus)* and two in paddlefish (*Polyodon spathula*) (Supplementary Table S4). These findings support a working hypothesis that a novel form, designated here as AHR4 and expressed as a closely related pair similar to but distinct from *Ahr1* and *Ahr2*, is expressed in at least a limited cluster of related species (Hahn et al., 2017). In this regard, it resembles AHR3 from the spiny dogfish (*Squalus ananthias*) (Hahn et al., 2017), but it is equally distinct from AHR 1–3 in the latter (Table S4).

**Fig. 8:**
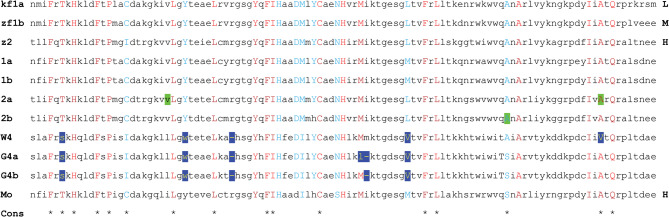
Alignment of ligand binding domains from present work (**1a**, **1b**, **2a**, **2b**, **W4**, **G4a**, **G4b**) for white sturgeon compared to zebrafish (D. rerio) zfAHR2 (**z2**, high affinity for TCDD), C57BL/6J mouse (M. musculus) mAHR (**Mo**, high affinity), zebrafish (D. rerio) zfAHR1b (**zf1b**, medium affinity) and killifish (F. heteroclitus) AHR1a (**kf1a**, low affinity). Residues modeled for **Mo** and **z2** as internal to the binding cavity are shown in capital letters as red if they are the same or blue if they differ between **Mo** and **z2**. In some cases, the residues match but one may not be modeled as internal and are shown in black as lower case. Residues conserved (**Cons**) in mammalian domains of high affinity are indicated by asterisks in the bottom line. Modeling results and alignments shown for mouse and zebrafish are those reported^30^. The amino acid sequences in green and white sturgeon AHR1 and AHR2 are identical except for the residues highlighted in green. In **2a**, these are, in order, i and v in green sturgeon; in **2b**, S in green sturgeon. Residues in white (W) and green (G) AHR4 differing from those in red or blue type are highlighted in dark blue. Capital letters in bold on the right margin indicate relative binding affinity (L, low; M, medium; H, high)^30^.

An important feature of an AHR is the affinity of its ligand binding domain (LBD) for small molecules that affect its function. Homology modeling of the AHR LBD has permitted identification of critical amino acid residues for high affinity TCDD binding^30^. LBD domains in the sturgeon sequences were aligned for comparison with high affinity mouse and zebrafish forms. The residues projecting into the ligand binding region are proposed to be major influences in TCDD binding affinity. According to modeling, the LBD in the mouse and zebrafish differ slightly in shape, accounting for small differences in critical residues. However, close inspection of the amino acid sequences in the LBD (Table S5 and Fig. 8) indicate that residues other than those identified as projecting into the binding region must influence the affinity.

The alignments with white sturgeon (Fig. 8) show that critical residues are conserved in sturgeon LBDs despite substantial differences in overall sequence identities (Table S6). Where the mouse and fish high affinity forms differ at these locations, the sturgeon forms match either mouse (Mo, 27 total) or zebrafish (z2, 25 total) critical sites. By this criterion, AHR4 differed in four critical sites from Mo and z2 and exhibited a one residue deletion. Green sturgeon AHR4a differed at 4 residues and AHR4b at 3 residues at those sites, each with two deletions. Overall, this analysis is consistent with sturgeon AHR1 and AHR2 having high affinity for TCDD, but AHR4 having lower affinity.

Similar to its expression in human keratinocytes, *Cyp1A* was expressed at low levels in the cultured cells in the absence of added AHR ligand. By contrast, *Cyp3A* was expressed at much higher levels. Two culture lines (GSA, WSM) expressed it at levels similar to *FlnA*, while one (GSI) expressed it at an order of magnitude higher level. This behavior also resembles expression of *CYP3A4 and CYP2A5* in human epidermal keratinocytes (Makihara et al., 2024). Both features are of potential importance for studying toxic responses of the cells to environmental chemicals.

## Discussion

The skin and mucosae of white sturgeon evaluated here were similar to comparable anatomic sites on the Yangtze sturgeon (*A. dabryanus*)^16^. All the regions examined in the integument were covered by a thick layer of epithelial cells that went through a terminal differentiation pattern similar to that in mammals without cornifying. However, each site had mucus producing cells in various distribution patterns and densities.

Cultivation of epithelial cells appeared routine in this work from a variety of sites in sturgeon, although the cells grew slowly and, in our experience, had to be grown at first with ciprofloxicin and amphotericin B to prevent contamination by micro-organisms. Microscopically, at low magnification, the cell lines from the various sites showed minor distinguishing features insufficient for certainty of identification. The similarity was surprising even for cells from such different sites as upper palate and ampullae of Lorenzini, the latter a region of electrosensory and mucus secreting cells embedded in a stratified epithelium^31^. Exceptions were cultures of esophageal cells, which secreted large amounts of mucus evident by the viscosity of the culture medium. The focus of the work was on optimizing growth of several strains from different anatomic sites to study their properties and suitability for exploration of toxic mechanisms. Extension to cells and, if necessary, explants from other critical epithelia such as the gills could be an advantageous approach to identify critical target sites.

Cornified anatomic structures in fish have long been documented^32^. Among teleosts, these can include horny projections (unculi) arising from single cells^33^breeding tubercles and contact organs^34^. Sturgeon can display “keratinized” or cornified spines on the skin^16^ that show some resemblance to the horny teeth of lamprey^35^ and hagfish^36^. Such observations indicate the existence of a regulatory system for cornification in such species, but how similar it is to that in human keratinocytes^37^and whether it can be perturbed by endocrine disruption, remains to be examined. Substantial keratin expression is seen in sturgeon epithelial cells in the present work, as in the case of lake sturgeon larvae^38^. This is consistent with keratin cytoskeletons being observed in various internal organs of fish, including sturgeon^39^and mammals^40^a characteristic of epithelial cells.

In mammals such as the rat^41^, expression of TGM1 is a feature almost exclusively of keratinocytes. *TGM1* is found throughout the vertebrate genomes^42^ and to include a membrane-binding cysteine cluster, in human^43^, tilapia and other teleosts^19^ and in sturgeon^12^. TGM1 and sulfhydryl oxidase (QSOX1) have been proposed as critical contributors to the process of cornification^44^. The cultured sturgeon epithelial cells have sufficient levels of TGM1 to form cross-linked envelope-like structures upon ionophore treatment^12^, a phenomenon also seen in cultured tilapia lip cells^19^, but they do not make envelopes in culture spontaneously, unlike mammalian keratinocytes. Present results show that QSOX1 expression is quite low in cultured sturgeon cells. Whether it is expressed at higher levels and how it participates in a regulatory process in anatomical regions of fish that become cornified is unknown, as is the trigger for TGM1 activation. Similarly, clear evidence for a stratum granulosum, which may be critical for cornification, was not observed. Regardless, the lack in fish of proteins encoded in the epidermal differentiation complex of mammals^45^which are incorporated into cornified structures, could distinguish these structures in fish and mammals.

Our previous work showed that epithelial cultures from the rim of white sturgeon mouth expressed TCDD-inducible *CYP1A*^12^. In the present work, TCDD was more potent in the cultured white and green sturgeon epithelial cells (EC50 *≤* 0.1 nM) than reported in white sturgeon liver explants (EC50 1 nM)^9^. The sensitivity of the cells to TCDD toxicity (in the absence of Y27632) showed a similar concentration dependence for EROD induction. The novel finding of toxicity in epithelial cells derived from several anatomic sites raises the possibility that, at least in mature sturgeon, sensitive target sites could be more general and include the integument. That previously observed signs of TCDD toxicity are similar or identical in fish of widely different sensitivities suggests the mechanism of action is the same^46^but further work will be needed to determine whether the novel AHR toxicity pathway in zebrafish^47^a much less sensitive species, also pertains to sturgeon integument.

Mice are well known to have naturally occurring polymorphisms of a single *Ahr* locus that influence TCDD toxicity^48^. By contrast, fish express several genes encoding AHR, only some of which bind TCDD and mediate toxic effects^49^. Homology modeling suggests both sturgeon AHR1 and AHR2 have high affinity for TCDD binding and rationalizes the greater potency of TCDD in white compared to lake sturgeon^50^. Both receptor forms could mediate toxic effects, as originally suggested^29^, though not necessarily by the same pathway. While ligand binding ability is not sufficient to yield toxic effects, which require receptor interactions with other proteins and transcriptional endpoints that could be altered by drift in receptor amino acid sequence, transfection experiments indicate both receptors are fully functional^51^. Further investigation of *Ahr* evolution using sturgeon and investigation of the ligand binding ability and functionality of *Ahr4* appear warranted.

How well cells in culture mimic those in the tissues of origin in vivo is a frequent consideration in their use for studying mechanisms of action and for monitoring environmental exposures. Here the slow genetic drift in sturgeon in vivo suggests the cells in culture may depart more slowly from normal than those from other species with faster genetic drift. In any case, the properties of these cells constitute a valuable culture model. Their expression of functional AHR could be applied in high throughput assays to monitor pollution containing EROD-inducing compounds. The remarkable ability of the rho kinase inhibitor Y27632, commonly used to delay senescence in keratinocytes^52,53^ to prevent TCDD toxicity may help understand the toxic pathway and shed light on the dramatic differences in species sensitivity. This phenomenon may be analogous to the antagonism of TCDD-induced growth suppression in human epidermal cells by epidermal growth factor^54^. Y27632 protects against senescence and apoptosis induced by various means^55,56^ and partially against toxicity from some chemical treatments^57^. However, the finding that this pharmaceutical acts beyond rho kinase inhibition^58^ will complicate elucidating responsible signaling pathways. Moreover, the observation of *Cyp3A* expression in the sturgeon cells offers the opportunity to explore the substrate specificity of this fish enzyme and consequences of exposure to pollutants that are its substrates or affect its activity.

## Electronic supplementary material

Below is the link to the electronic supplementary material.

Supplementary Material 1

## Data Availability

Original sequencing data are available in Gene Expression Omnibus at https://www.ncbi.nlm.nih.gov/geo/, reference number GSE291000. The nucleotide sequences of PacBio transcripts of the genes examined by qPCR and their amino acid translations for green and white sturgeon have been submitted to GenBank (Table S7). Inquiries about the green sturgeon genomic scaffold can be directed to Andrea Schreier (amdrauch@ucdavis.edu).
