# Supplementary material for: An epithelial cell culture model for sturgeon integument responds sensitively to 2,3,7,8-tetrachlorodibenzo-p-dioxin exposure
Source: Sci Rep. 2025 Jul 24;15:26875. doi: 10.1038/s41598-025-12299-7 (PMC12290088; doi:10.1038/s41598-025-12299-7)
Supplement: Supplementary file 1 — Supplementary Material 1 [file 41598_2025_12299_MOESM1_ESM.pdf]

An Epithelial Cell Culture Model for Sturgeon Integument Responds Sensitively to 2,3,7,8-Tetrachlorodibenzo-p-dioxin Exposure

Sumi Nechat, Melina Shadi, Andrea D. Schreier,
Nann A. Fangue, John P. Sundberg, and Robert H. Rice

Supplementary Material

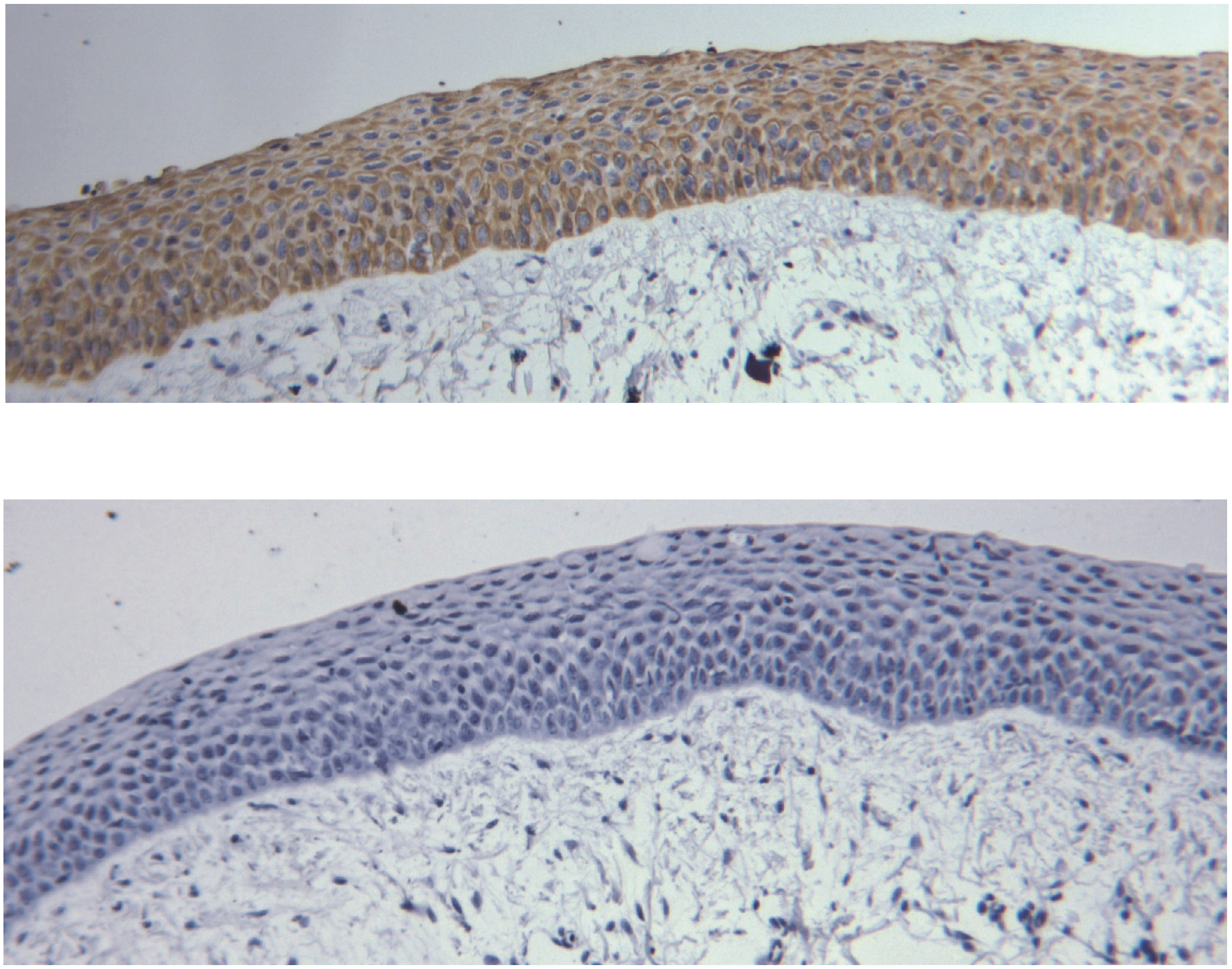

Supplementary Figure S1. Sections of oral epithelium stained with (A) or without (B) anti-pan-cytokeratin monoclonal antibodies. Both sections were counter stained with hematoxylin.

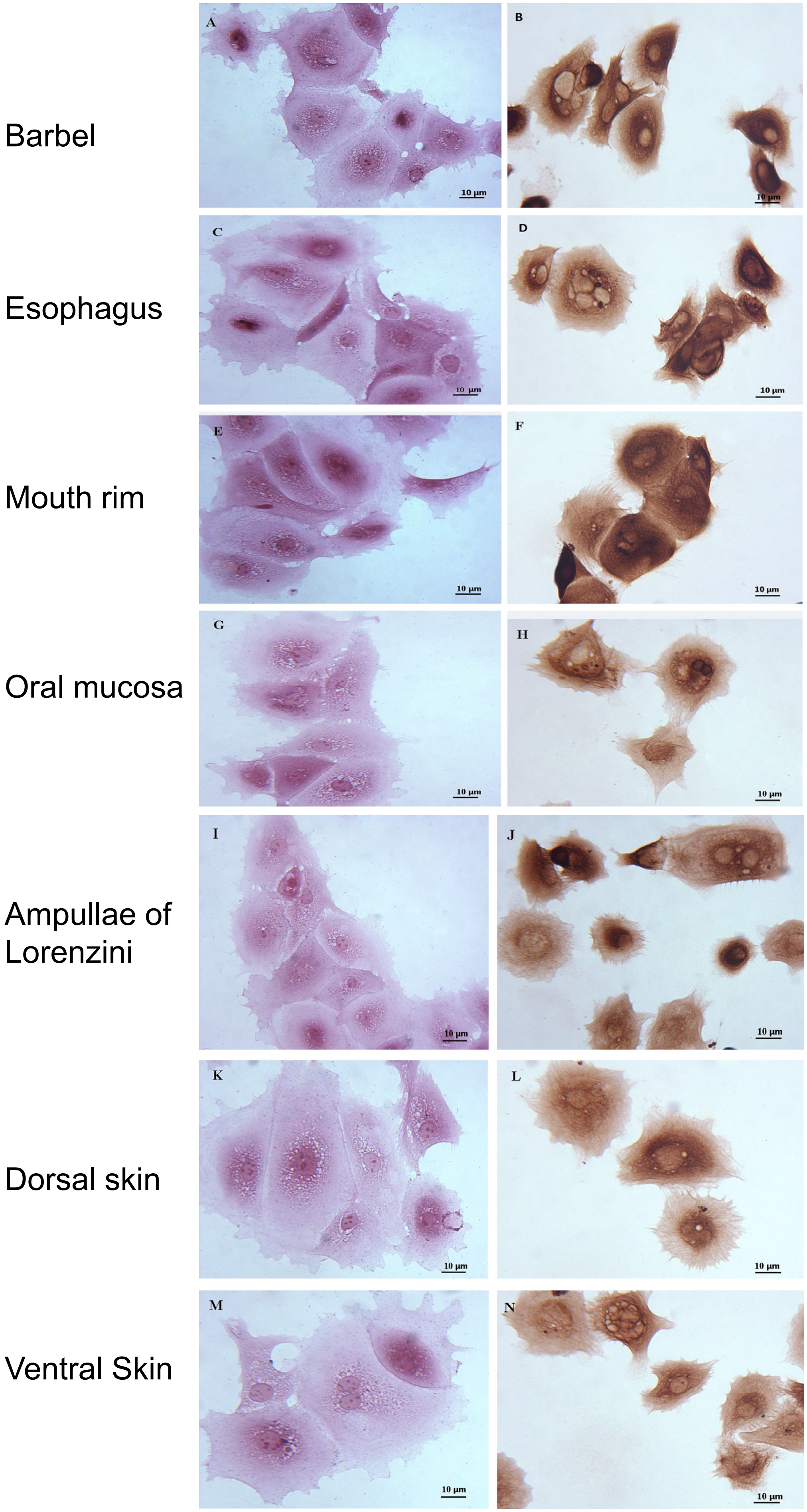

Supplementary Figure S2. Cells cultured from the indicated tissue sources were either stained with rhodanile blue (left column) or immunostained with anti-pancytokeratin monoclonal antibodies (right column).

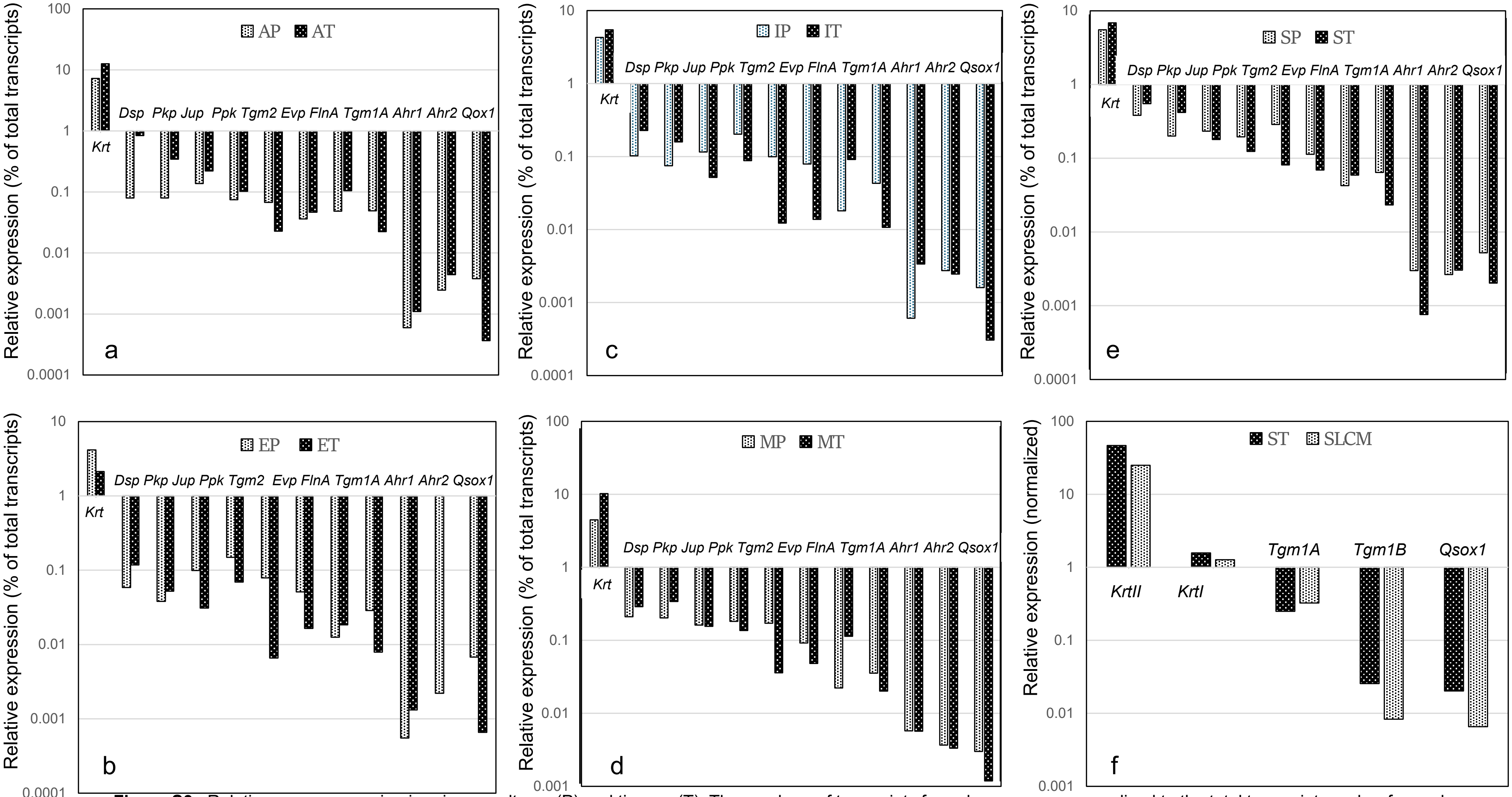

Figure S3. Relative gene expression in primary cultures (P) and tissues (T). The numbers of transcripts for a dozen genes, normalized to the total transcript number for each sample, are shown in **a** (ampullae of Lorenzini), **b** (esophagus), **c** (upper palate), **d** (rim of protrusible mouth) and **e** (skin). Relative transcript levels determined by qPCR are shown in **f** for skin tissue (T) obtained by laser capture microscopy for *KrtII, KrtI*, *Tgm1A, Tgm1B* and *Qsox1*, all normalized to one for the root mean square of *Dsp* and *FnlA*.

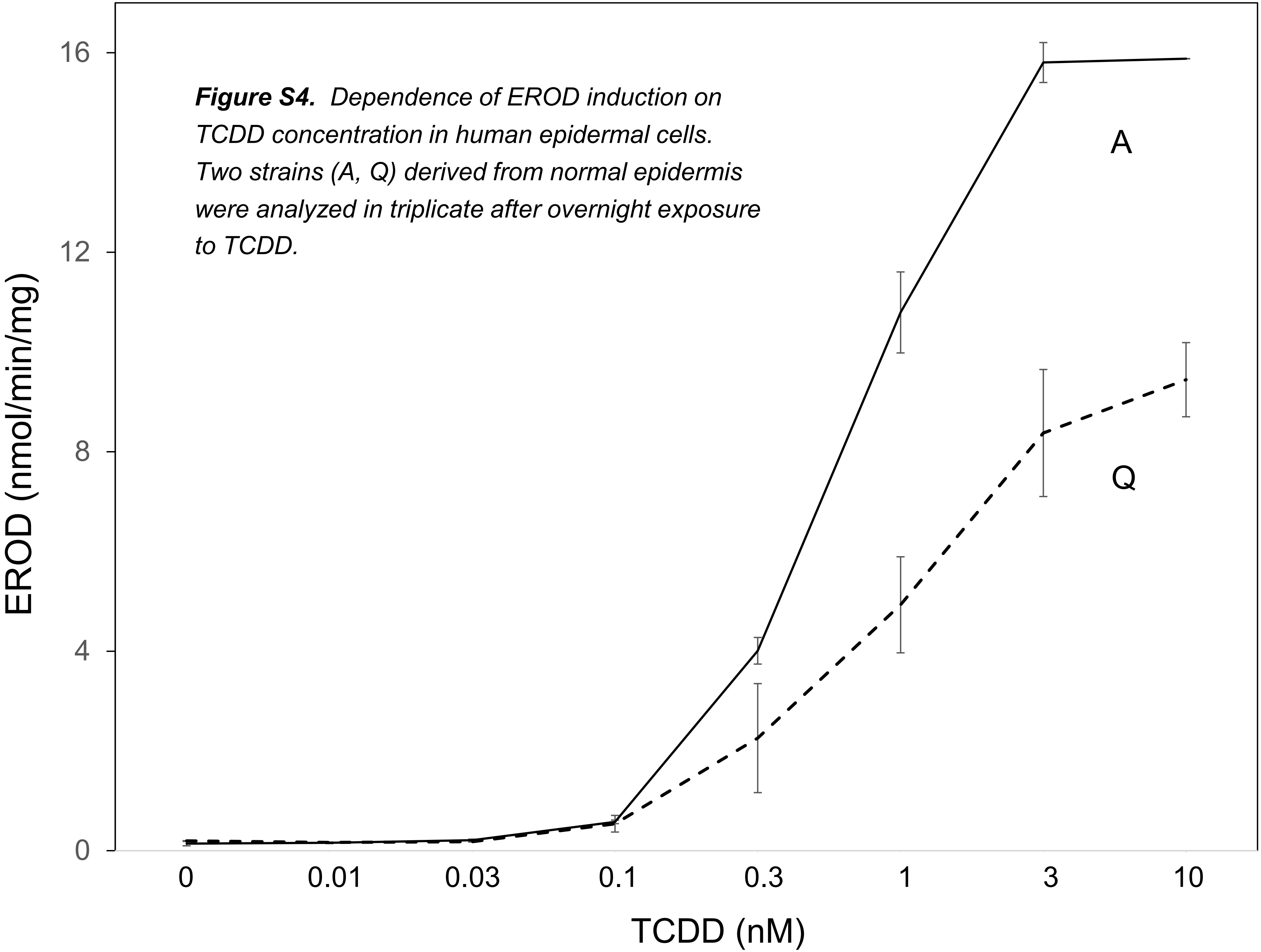

Figure S4. *Dependence of EROD induction on TCDD concentration in human epidermal cells. Two strains (A, Q) derived from normal epidermis were analyzed in triplicate after overnight exposure to TCDD.*

Supplemental Figure S5. Y27632 suppression of TCDD (1 nM) toxicity in GSA cells treated for 16 days. The dashed line is a measure of cell protein at the start of treatment. C, control (no addition); E, EGF; Y, Y27632. Like EGF, insulin was ineffective in suppressing the observed toxicity. *, significantly different from control ($p = 4x10^{-7}$).

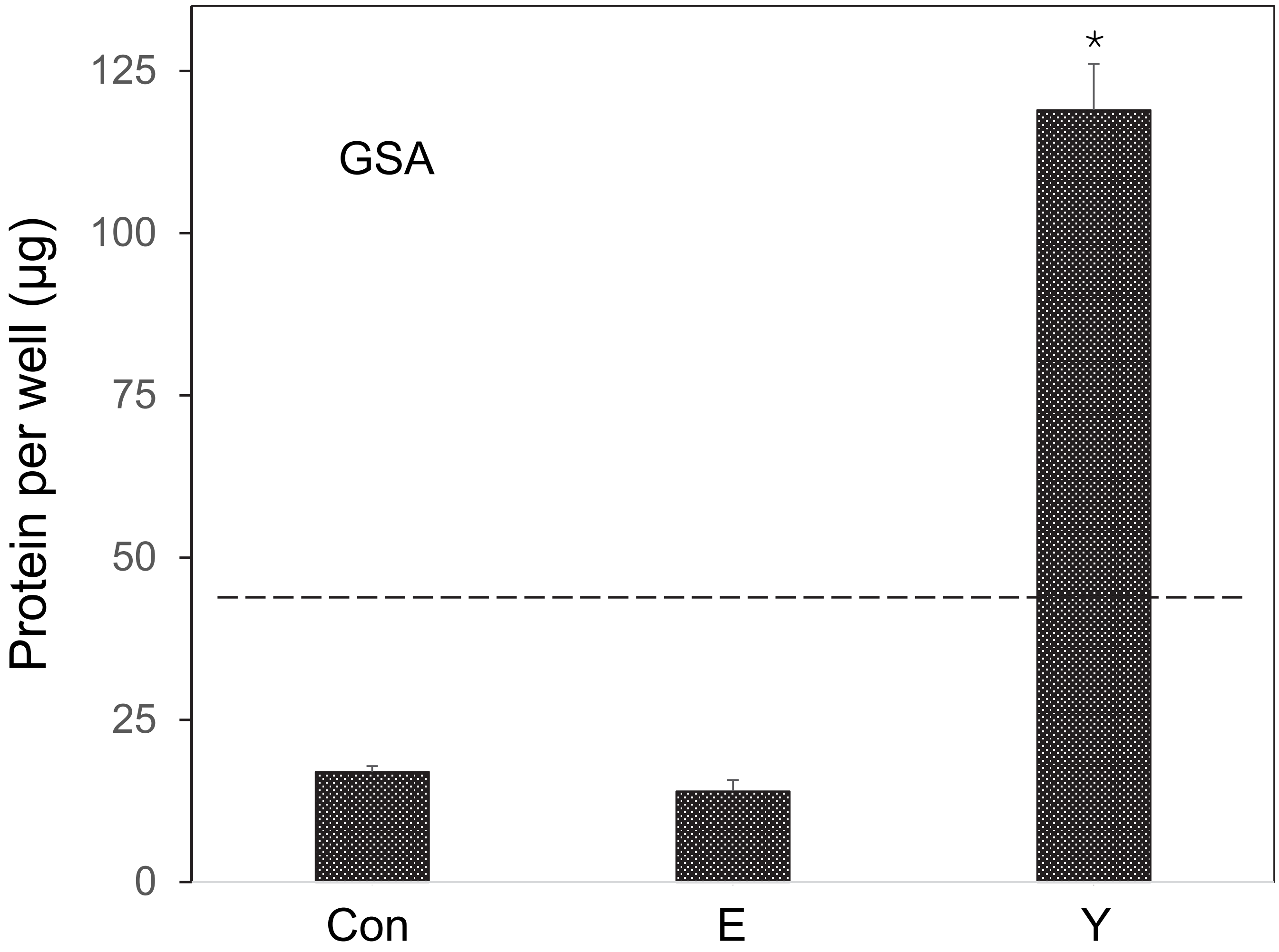

Supplementary Figure S6. Shown are the original (uncropped and unedited) images used to compose Figure 4 b and c.

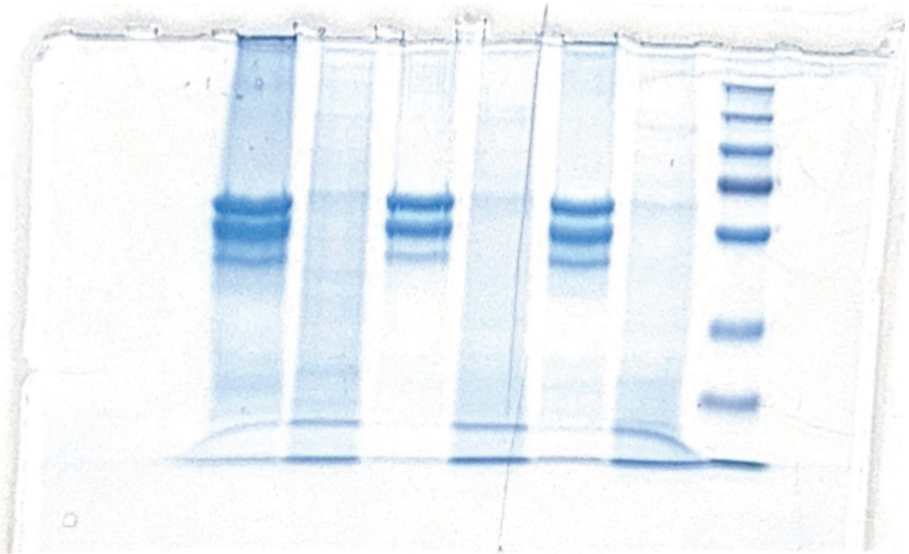

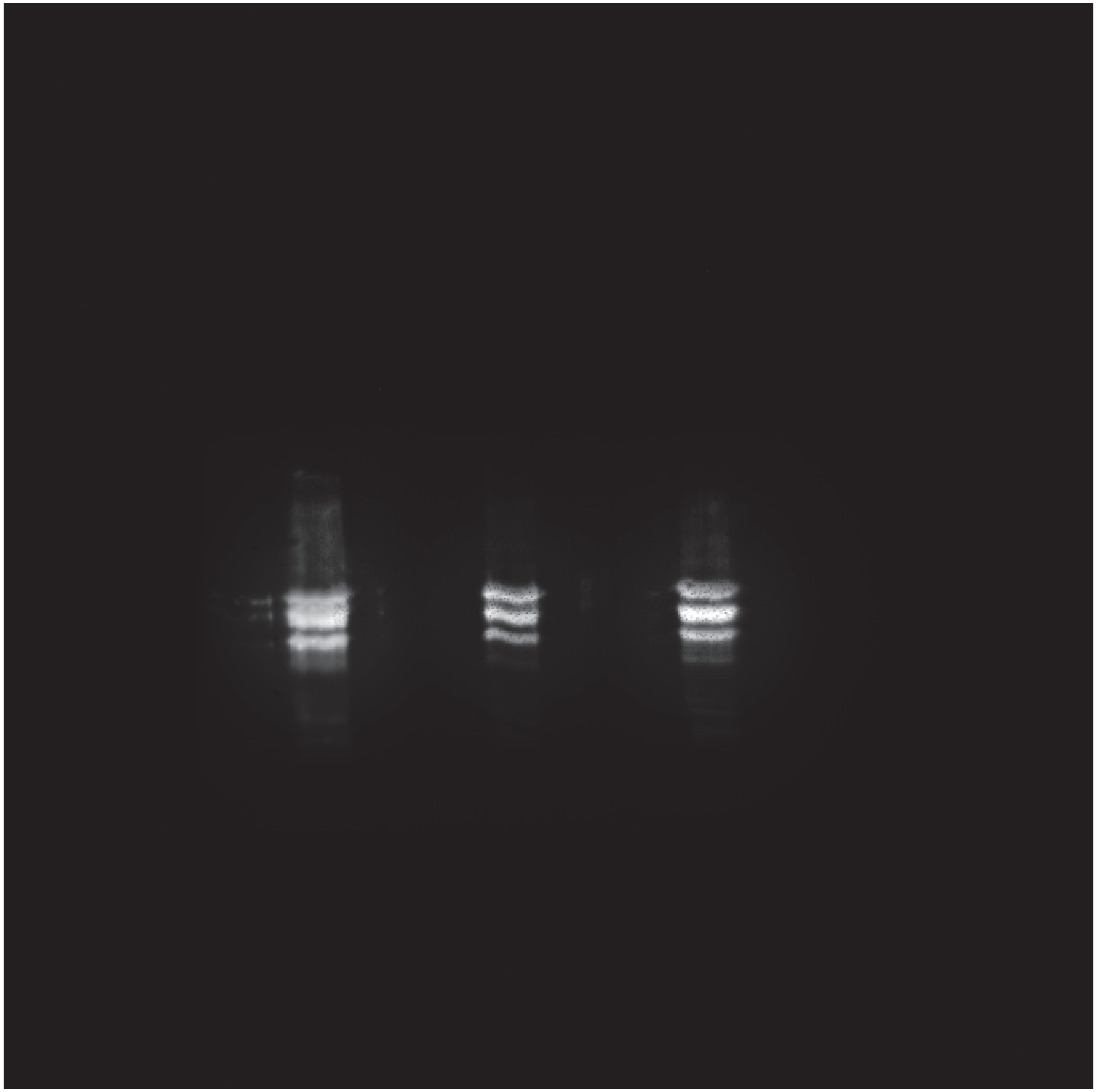

Table S1. Sequences of primers and reporters provided by Applied Biosystems in the custom Taqman gene expression assays.

Gene	Forward	Reverse	Reporter
Ahr1	GCCCTCAGGATAGTATGATTACTTCAC	TTCATCTGATTTACTTGATTCATACAAGTAGA	TCTGCCGGAAACTC
Ahr2	TTCAAAGAGCAATGCAAACCCAA	CCATTCAGAAGTTGGCCTTCAAAT	CCAACCAGATGGATTCC
Ahr4	GGCAAATGAGCAGCTCGTT	AGCATGGTAAGTAAAGCAGAATGGT	CTCCCACATCAAACTC
Cyp1a	CCGCAGTGAAGTCTTCCTCTT	GGGTGAGGTCCAGCTTCTG	CTGGCTGTGCTCCTGC
Cyp3a	GCGATGGAGTACCTGGACATG	CAGGGCATAGGTGGGTATCATAAC	CAAGCCGTGGTGCTGC
Dsp	CGTCTGTTAGAAGCTCAGATTGCA	ACTTTTAAGCGATGACTAGCTTTTGG	CCGGTGGAATCATTG
FlnA	ACCCAGTAAAGCTGAGATCTCCTT	CTCCTGGACGATGTACGAAACC	CATCCTTGCGATCCTC
KrtI	CGCTATCAGCTCCTTCACTTCTT	GAGCCTACCCTGTGTTGGG	CTGCGTCACCATGTCTG
KrtII	CTCCCTGGGCAATGACAAGA	GTCCTCCACCATGTTCTGCATATTA	ATCGCTCTCCAGCTTCA
Qsox1	AGACAGTGCTGGACAATGAAGTG	CACTGTGAGCAGGTGGAAGAG	CTGGCAGGACACCCAC
Tgm1A	CAGACGCCCAAGAGAATCAAC	TCTGTCAGGGTGATTGTAGAGTGA	ATCGGCGATGTGGCACG
Tgm1B	CGCGGTGATCAGCTTGGA	TCAGGGTGAGTGTGGAGTGA	ACGTCGCCAATGTTG

Table S2. % Identities among AHR forms identified by PacBio sequencing.

Green	1b	2a	2b	4a	4b
1a	82	54	54	45	46
1b		55	54	46	47
2a			89	49	49
2b				48	50
4a					89

White	1b	2a	2b	4
1a	83	55	55	46
1b		55	56	46
2a			90	49
2b				49

Table S3. % Identities among AHR forms in this work with those of white sturgeon (W) and *Acipenser fulvescens* (F)[15].

Green	W1*	W2†	F1‡	F2§
1a	93	54	93	54
1b	86	55	93	55
2a	55	93	54	90
2b	54	94	54	97
4a	45	49	46	48
4b	47	49	47	49

White	W1*	W2†	F1‡	F2§
1a	93	55	93	54
1b	96	55	97	55
2a	55	94	55	90
2b	55	96	55	98
4	46	49	47	49

GenBank Accession numbers: * KJ420394.1,
† KJ420395.1, ‡ KM236089.1, § KM245041.1

Table S4. % Identities in comparisons of putative AHR4 forms from green and white sturgeons with those of *Acipenser oxyrinchus oxyrinchus* (Ao; [a]KAK1162081.1),*Acipenser ruthenus* (Ar; [a]XP_033896483.2, [b]XP_033898209.1), *Polyodon spathula* (Ps; [a]XP_041078280.1, [b]XP_041125165.1) and *Squalus acanthias* (Sa; [a]AFR24092.1, [b]AFR24093.1, [c]AFR24094.1).

	Ao[a]	Ar[a]	Ar[b]	Ps[a]	Ps[b]	Sa1[a]	Sa2a[b]	Sa3[c]
Green 4a	94	96	89	88	88	52	49	49
Green 4b	89	88	96	90	90	50	49	47
White 4	95	100	82	89	89	47	49	48

Table S5. % Identities in comparisons among sturgeon ligand binding domains in this work (column 1) to AHR1 and AHR2 given by Doering et al (2014) for white sturgeon. In that work, AHR1 and AHR2 were 76% identical.

Green	AHR1	AHR2
AHR1a	98	74
AHR1b	99	73
AHR2a	75	97
AHR2b	73	98
AHR4a	55	53
AHR4b	55	52

White	AHR1	AHR2
AHR1a	99	73
AHR1b	99	74
AHR2a	76	99
AHR2b	73	98
AHR4	57	54

Table S6. % Identities among sturgeon AHR forms in present work compared to zAHR1b (NP_001019987) and zAHR2 (NP_571339) in zebrafish and mAHR (NP_038492.1) in C57BL/6 mice.

White	zAHR1b	zAHR2	mAHR
AHR1a	55	54	51
AHR1b	55	53	54
AHR2a	54	71	51
AHR2b	53	69	51
AHR4	52	49	51

Green	zAHR1b	zAHR2	mAHR
AHR1a	59	54	52
AHR1b	59	54	48
AHR2a	54	70	60
AHR2b	53	70	52
AHR4a	52	49	50
AHR4b	53	54	51

Table S7. GenBank accession numbers for PacBio nucleotide sequences derived in this work:

BankIt2964945 white_AHR1a	PV738898
BankIt2964945 white_AHR1b	PV738899
BankIt2964945 white_AHR2a	PV738900
BankIt2964945 white_AHR2b	PV738901
BankIt2964945 white_AHR4	PV738902
BankIt2964945 Green_AHR2b	PV738903
BankIt2964945 Green_AHR2a	PV738904
BankIt2964945 Green_AHR1b	PV738905
BankIt2964945 Green_AHR1a	PV738906
BankIt2964945 Green_AHR4a	PV738907
BankIt2964945 Green_AHR4b	PV738908
BankIt2965021 Green_DSP-like_X3	PV738909
BankIt2965021 Green_DSP-like_X2	PV738910
BankIt2965021 White_DSP-like_X1	PV738911
BankIt2965021 White_DSP-like_X2	PV738912
BankIt2966092 GREEN_KRT50kDa-like	PV738913
BankIt2966092 WHITE_KRT50kDa-like	PV738914
BankIt2966092 GREEN_KRT13-like	PV738915
BankIt2966092 WHITE_KRT13-like	PV738916
BankIt2966092 GREEN_KRT19-like_Partial_20745.23	PV738917
BankIt2966092 WHITE_KRT19-like_20745.23	PV738918
BankIt2966092 GREEN_KRT19-like_Partial_20748.62	PV738919
BankIt2966092 WHITE_KRT19-like_20748.38	PV738920
BankIt2966092 GREEN_KRT13-like_22698.13	PV738921
BankIt2966092 WHITE_KRT13-like_22698.17	PV738922
BankIt2966092 GREEN_KRT19-like_22701.17	PV738923
BankIt2966092 WHITE_KRT19	PV738924
BankIt2966092 GREEN_KRT50kDa-like_25257.5	PV738925
BankIt2966092 WHITE_KRT50kDa-like_25257.16	PV738926
BankIt2966092 GREEN_KRT8-like_25399.18	PV738927
BankIt2966092 WHITE_KRT8-like_25399.216	PV738928
BankIt2966092 GREEN_KRT8-like_25558.105	PV738929
BankIt2966092 WHITE_KRT8-like_25558.1011	PV738930
